# Decadal Evolution of Atmospheric Sulfate and Nitrate and Its Potential Link to Energy Restructuring: A Case Study in Guangdong Province, China

**DOI:** 10.1002/gch2.70108

**Published:** 2026-04-25

**Authors:** Haohao Wang, Qing Chen, Kangan Shu, Jinqing Luo, Liu Yang

**Affiliations:** ^1^ Guangdong Power Exchange Center Co., LTD Guangzhou China

**Keywords:** aerosol acidity, energy restructuring, NO_x_ and SO_2_, secondary inorganic aerosols

## Abstract

The global shift toward renewable energy and the wide adoption of electric vehicles (EVs) are expected to mitigate a series of global environmental concerns, while few studies have focused on their contribution to reducing PM_2.5_ concentrations. This study investigates the temporal evolution of secondary inorganic aerosols (SAIs, sulfate and nitrate) and their gaseous precursors (SO_2_ and NO_2_) in Guangdong from 2014 to 2024. By integrating multisource datasets, including air pollutant monitoring, aerosol chemical composition, energy use, and EV statistics, this study quantifies the contributions of coal consumption reduction and clean energy growth to SO_2_ mitigation, and further examines the association between transportation electrification and NO_2_ decline. Meanwhile, the changes in the sulfate‐to‐nitrate ratio and aerosol acidity are also analyzed, highlighting the role of evolving atmospheric chemistry in shaping PM_2.5_ composition and guiding future emission control strategies.

## Graphical abstract

Energy transition and transportation electrification restructure emissions and shift aerosol chemistry in Guangdong. Concurrent declines in sulfate and nitrate indicate reduced secondary inorganic aerosol (SIA) formation, while evolving chemical regimes modulate PM_2.5_ composition. These findings highlight the importance of coupling emission control with atmospheric chemistry.

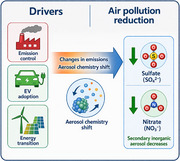

## 1 Introduction

Air pollution, as a globally important environmental challenge, poses significant threats to public health, ecosystems, and economic development [1, 2]. Fine particulate matter (PM_2.5_) is of particular concern due to its ability to penetrate deep into the respiratory system [3] and its complex chemical composition. Among its major components, sulfate (SO_4_^2−^) and nitrate (NO_3_^−^) aerosols have long been recognized as dominant contributors to PM_2.5_ mass [4], formed primarily through the atmospheric oxidation of sulfur dioxide (SO_2_) and nitrogen oxides (NO_x_), respectively [5, 6, 7].

The emission sources of SO_2_ and NO_x_ are closely linked to energy and transportation structures [8, 9, 10, 11]. Previous studies have shown that the power sector is an important source associated with sulfate and nitrate formation [12, 13, 14], while road transportation contributes substantially to NO_x_ emissions in densely populated and highly motorized regions [15]. Therefore, transitions in the energy and transportation sectors are expected to have differentiated influences on ambient SO_2_ and NO_2_ [16, 17].

Over the past decade, China has undergone substantial energy restructuring and transportation electrification. Although previous studies have documented long‐term changes in air pollutant concentrations and, in some cases, PM_2_._5_ chemical composition [18, 19, 20], integrated analyses that explicitly link these trends to concurrent structural changes in the energy and transportation sectors remain limited. More importantly, comprehensive studies that simultaneously consider both secondary particulate components and their gaseous precursors (SO_2_ and NO_2_) are still lacking. Because sulfate and nitrate concentrations are jointly influenced by precursor emissions and atmospheric transformation processes, considering only precursor gases or only particulate species cannot provide a complete understanding of the chemical evolution of fine particulate matter [21, 22].

In this context, Guangdong Province serves as an ideal case study. As one of the most economically developed and densely populated regions in China, Guangdong has experienced rapid energy transition and transportation electrification over the past decade [23, 24, 25, 26]. These transitions, coupled with its historically high emissions of SO_2_ and NO_x_ [27, 28], make Guangdong highly representative of broader trends occurring in other urbanized and industrialized regions across the country. Therefore, investigating the evolution of secondary inorganic aerosols (SIAs) and their precursors in Guangdong can provide valuable insights into how air quality responds to structural changes under the dual pressures of pollution control and low‐carbon development.

In this study, we analyzed the long‐term evolution of SIAs (especially sulfate and nitrate) and their gaseous precursors (SO_2_ and NO_2_) in Guangdong Province from 2014 to 2024. By integrating multisource datasets, including ground‐based pollutant measurements, aerosol chemical composition data, meteorological reanalysis, and statistical records on energy consumption and vehicle electrification, we investigated long‐term pollutant trends and the responses of aerosol composition to changes in precursor emissions. We further quantified the respective contributions of coal consumption restructuring and clean energy development to SO_2_ mitigation.

## 2 Datasets

### 2.1 Air Pollutant Concentrations

Air pollutant data were obtained from the National Urban Air Quality Real‐Time Release Platform operated by the China National Environmental Monitoring Centre (CNEMC, https://air.cnemc.cn:18007/), covering the period from May 1, 2014, to December 31, 2024, for 21 prefecture‐level cities in Guangdong Province. The monitored pollutants include all regulated pollutants: PM_2.5_, PM_10_, SO_2_, NO_2_, CO, and O_3_. These measurements were conducted within the national ambient air quality monitoring network in accordance with relevant Chinese national monitoring standards for continuous automated monitoring of particulate and gaseous pollutants (HJ 653–2013/HJ 653–2021; HJ 654–2013), using standardized instruments and protocols. For each city, the pollutant concentrations represent the average across all monitoring stations, while the provincial average is calculated as the mean of the city‐level averages. Observations are available at an hourly resolution and were aggregated into monthly and annual averages for analysis. The original monitoring data had undergone routine quality assurance and quality control procedures by the data provider, and additional screening was applied in this study to remove missing and invalid records before aggregation. Monitoring operation and QA/QC followed the relevant Chinese technical specifications for continuous automated monitoring of particulate and gaseous pollutants (HJ 817–2018; HJ 818–2018) [29].

### 2.2 Aerosol Composition

The aerosol chemical composition data used in this study were obtained from the Tracking Air Pollution in China (TAP) dataset (http://tapdata.org), developed under the leadership of Tsinghua University [30]. The TAP dataset integrates satellite observations, ground‐based measurements, meteorological reanalysis data, and emission inventories, and applies chemical transport modeling combined with data assimilation techniques to produce high‐resolution regional estimates [31]. Through comparison with ground‐based observations, the reliability of the TAP dataset has been evaluated, demonstrating good agreement between satellite‐derived estimates of PM_2.5_ chemical components and in situ measurements, with correlation coefficients (*R*) ranging from 0.56 to 0.81 across different species [32]. Monthly mean concentrations of fine particulate matter (PM_2.5_) and its major components, including SO_4_^2−^, NO_3_^−^, NH_4_^+^, black carbon (BC), and organic matter (OM), in Guangdong Province from January 2014 to December 2024 were used in this study. The sulfur oxidation ratio (SOR) and nitrogen oxidation ratio (NOR) were calculated as SOR = [SO_4_^2−^]/([SO_4_^2−^] + [SO_2_]) and NOR = [NO_3_^−^]/([NO_3_^−^] + [NO_2_]), respectively, where brackets denote molar concentrations.

Aerosol acidity in this study is represented by aerosol pH estimated using the Extended Aerosol Inorganics Model (E‐AIM‐II) in forward mode under the metastable assumption. The model inputs included sulfate, nitrate, and ammonium concentrations, together with ambient temperature and relative humidity. The estimation procedure followed that described by Cui et al. (2025) [33].

### 2.3 Energy Consumption and Vehicle‐Related Data

Energy consumption and power generation data for Guangdong Province were obtained from the China Energy Statistical Yearbook published by the National Bureau of Statistics (https://www.stats.gov.cn). The dataset covers: (1) annual consumption of primary energy sources, including raw coal, crude oil, electricity, and natural gas, across all usage sectors, with all quantities converted into kilograms of standard coal equivalents for comparison; (2) coal consumption for major sectors, including power generation, industrial processes, and transportation and (3) the composition of power generation, distinguishing between thermal power and clean energy sources such as hydropower, wind, nuclear, and solar energy. All energy‐related data were available from 2014 to 2022, as the most recent updates for 2023 and 2024 were not yet published.

Total vehicle numbers in each year were obtained from the Guangdong Statistical Yearbook (2014–2024). Data on electric vehicles (EVs) were sourced from the Annual Report on the Development of Electric Vehicle Charging Infrastructure in China, which is published annually by the China Electric Vehicle Charging Infrastructure Promotion Alliance (https://www.evcipa.org.cn). All energy‐related data were available from 2014 to 2022, as the most recent updates for 2023 and 2024 were not yet published. Because EV statistics for 2014–2016 were incomplete, EV‐related analyses in this study focus on the period from 2017 to 2024 [34].

### 2.4 Meteorological Data

Meteorological variables were obtained from the ERA5 reanalysis dataset developed by the European Centre for Medium‐Range Weather Forecasts (ECMWF), via the Copernicus Climate Data Store (https://cds.climate.copernicus.eu; last accessed February 27, 2025). ERA5 provides high‐resolution (0.25° × 0.25°) atmospheric variables using a 4D‐Var assimilation system [35, 36]. Extracted parameters include 2‐meter air temperature (*T*), dew point temperature (Td), 10‐meter zonal (u10) and meridional (v10) wind components, and total precipitation. Relative humidity (RH) was derived from T and Td. Data were spatially mapped to 21 city centers in Guangdong Province based on their geographic coordinates, and monthly averages were used in this study.

### 2.5 Linear Attribution Model

To quantify the contribution of sectoral coal consumption to the observed decline in SO_2_ concentrations, a linear attribution model was applied. In this approach, the annual average SO_2_ concentration was expressed as a linear combination of coal consumption in the power generation and industrial sectors:

(1) $$C\left(t\right)=\alpha \cdot E_{\mathrm{power}}\left(t\right)+\beta \cdot E_{\mathrm{industry}}\left(t\right)$$

where *C*(*t*) is the observed SO_2_ concentration in year *t*; *E*_power_(*t*) and *E*_industry_(*t*) represent the annual coal consumption for power generation and industrial sectors, respectively; α and β are the sector‐specific scaling factors, interpreted as relative emission intensities. The model assumes that the relative emission factor for each sector remains constant over time and therefore captures the relative contributions of changes in sectoral coal use rather than time‐varying emission controls or technology upgrades. The model parameters were fitted using annual data from 2014 to 2022.

## 3 Results and Discussion

### 3.1 Annual Trends in Air Pollutant Concentrations in Guangdong Province

**FIGURE 1:**
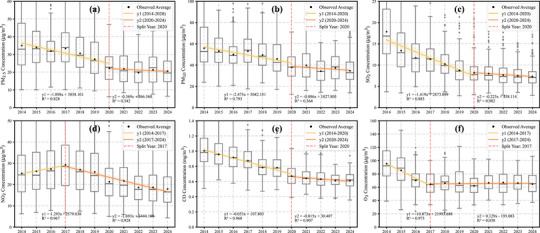
Annual trends of ambient air pollutants from 2014 to 2024 in Guangdong Province: (a) PM_2.5_, (b) PM_10_, (c) SO_2_, (d) NO_2_, (e) CO, and (f) O_3_.

Figure 1 presents the annual average concentrations of major air pollutants in Guangdong Province from 2014 to 2024. Overall, all major pollutants exhibited decreasing trends over the study period. The most notable decline was observed for SO_2_ (Figure 1c), with concentrations decreasing by approximately 60%, from 17.9 µg/m^3^ in 2014 to around 7 µg/m^3^ in 2024. PM_2.5_, PM_10_, and CO showed similar reduction rates of approximately 40%. In contrast, O_3_ and NO_2_ exhibited relatively smaller reductions of about 30%.

Between 2014 and 2024, PM_2.5_ and PM_10_ concentrations exhibited clear two‐phase declines, with rapid reductions before 2020 (−1.90 and −2.48 µg m^−3^ yr^−1^, respectively) and significantly slower rates thereafter (−0.37 and −0.89 µg m^−3^ yr^−1^). Figure 1c,e further illustrates that CO and SO_2_ followed similar patterns, with CO decreasing by −0.053 mg m^−3^ yr^−1^ before 2020 and −0.015 mg m^−3^ yr^−1^ after, while SO_2_ reductions slowed from −1.42 to −0.22 µg m^−3^ yr^−1^, suggesting that the rate of improvement slowed after 2020, despite continued reductions [37]. In contrast, Figure 1d shows that NO_2_ increased markedly from 2014 to 2017 (+1.29 µg m^−3^ yr^−1^) before shifting to a declining trend (−1.69 µg m^−3^ yr^−1^), reflecting strengthened mitigation efforts. The initial increase in NO_2_ until 2017 may reflect a delayed response to strengthened NO_x_ emission controls, with subsequent declines becoming evident as these measures took effect [38]. Figure 1f indicates that O_3_ exhibited a different trend, with concentrations decreasing sharply before 2017 (−10.87 µg m^−3^ yr^−1^) but stabilizing or slightly increasing thereafter (+0.13 µg m^−3^ yr^−1^), highlighting the complexity of ozone formation and its sensitivity to precursor emissions and meteorological variations [39].

Meteorological conditions were analyzed (Figure S1). Over the study period, temperature showed a slight increasing trend, while humidity, precipitation, and wind speed remained relatively stable with no clear long‐term trends. Overall, the key meteorological parameters remained broadly consistent throughout the study period, suggesting that large‐scale changes in meteorological conditions were not a dominant feature during these years. This relative meteorological stability suggests that the observed air quality improvements were less likely to be driven by large‐scale meteorological changes alone. Further analyses are still needed to disentangle the respective contributions of meteorological variability and emission changes.

### 3.2 Variation of Composition in PM_2.5_

**FIGURE 2:**
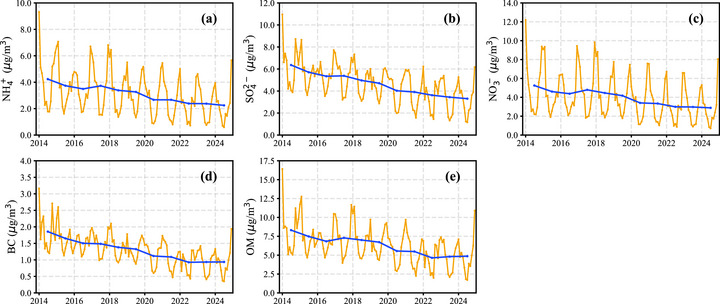
Temporal trends of major PM_2.5_ chemical components in Guangdong Province from 2014 to 2024. (a) Ammonium (NH_4_^+^); (b) sulfate (SO_4_^2−^); (c) nitrate (NO_3_^−^); (d) black carbon (BC); (e) organic matter (OM). Orange lines indicate monthly mean concentrations, and blue lines represent annual averages.

Figure 2 shows the temporal trends of individual PM_2.5_ components in Guangdong Province from 2014 to 2024. All major chemical species, including BC, ammonium (NH_4_^+^), nitrate (NO_3_^−^), OM, and sulfate (SO_4_^2−^), exhibited significant declining trends over the decade. The reduction rates for all components were relatively consistent, ranging between 41.5% and 49.5% (Figure 2). Further analysis revealed strong correlations among these components, with Pearson correlation coefficients (*r*) exceeding 0.95 for all pairs (Figure S2). Notably, sulfate and nitrate exhibited an especially high correlation, with an *r* value of 0.97, which is substantially higher than the correlation between their respective precursors, SO_2_ and NO_2_ (*r* = 0.63). This discrepancy may be attributed to the fact that SO_2_ and NO_2_ concentrations are primarily driven by emission sources, and their correlation reflects the differences in control measures targeting those sources [40]. In contrast, both sulfate and nitrate are secondary products formed through atmospheric oxidation processes [41]. Therefore, beyond their precursor concentrations, their levels are also influenced by common influencing factors, potentially explaining the stronger correlation observed between them.

Further analysis of the contributions to the overall PM_2.5_ decline reveals that SIAs, composed of sulfate, nitrate, and ammonium, collectively accounted for the largest share, contributing approximately 57.1% of the total PM_2.5_ mass reduction, consistent with previous report [38]. As illustrated in Figures 2a–c, within this group, sulfate contributed 23.6%, nitrate 18.2%, and ammonium 15.3%. OM also played a major role, accounting for 26.6% of the total decrease (Figure 2e), while black carbon (BC) contributed a relatively smaller share of 7.1% (Figure 2d). These results indicate that the overall PM_2.5_ improvement was primarily driven by the substantial reductions in both SIAs and organic aerosols, with SIAs being the single largest contributor [42]. This finding reinforces the importance of targeting precursor emissions such as SO_2_, NO_x_, and NH_3_, which drive secondary aerosol formation [43]. The quantified contributions also suggest that further air quality gains may increasingly depend on controlling more complex and diverse sources of organic aerosols and carbonaceous particles, especially as reductions in SIAs begin to plateau [44].

**FIGURE 3:**
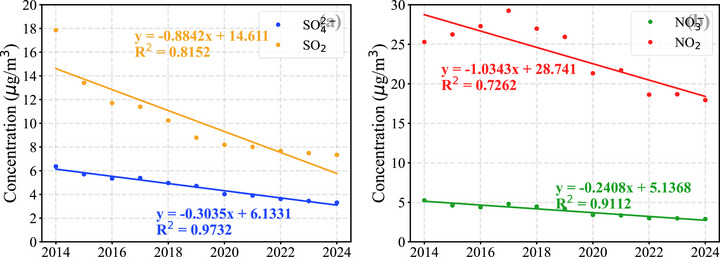
Comparison of decreasing trends between secondary inorganic aerosols and their precursors in Guangdong Province from 2014 to 2024. (a) SO_4_^2−^ and SO_2_; (b) NO_3_^−^ and NO_2_.

As shown in Figure 3, sulfate and nitrate concentrations declined substantially more slowly than their corresponding precursors. This discrepancy likely arises because SO_2_ and NO_2_ are directly emitted, whereas sulfate and nitrate are formed through more complex secondary processes in the atmosphere. As a result, their responses to precursor emission reductions may be delayed or less direct. The relationships between SO_2_ and sulfate, as well as between NO_2_ and nitrate, are therefore nonlinear and are better described by quadratic fits, as shown in Figure S3.

**FIGURE 4:**
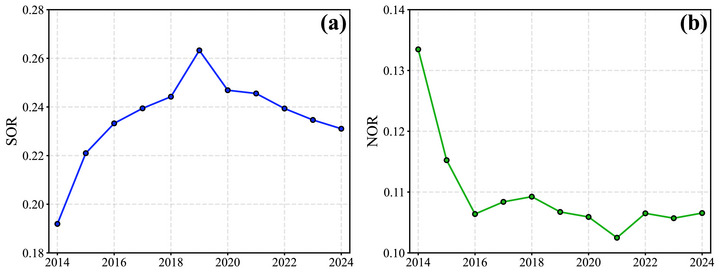
Annual trends of sulfur oxidation ratio (SOR; a) and nitrogen oxidation ratio (NOR; b) in Guangdong Province during 2014–2024.

Figure 4 shows that the SOR increased initially and then stabilized after 2018, whereas the NOR decreased before leveling off during the same period. Higher oxidation efficiency may partly offset the benefits of precursor emission reductions, thereby weakening control effectiveness for sulfate or nitrate. Conversely, lower oxidation efficiency may enhance the benefits of emission reductions [45]. Although both SOR and NOR reflect oxidation processes, their temporal trends diverge due to differences in their respective chemical pathways. For instance, Liu et al. (2020) found that NOR was significantly correlated with the product of [NO_2_]^2^ and [O_3_], suggesting a dependence on photochemical oxidants [22]. In contrast, the oxidation of SO_2_ is primarily governed by aqueous‐phase reactions involving H_2_O_2_ and is strongly influenced by ambient humidity.

These results suggest that the formation efficiency of SIAs is not solely determined by precursor availability, but also closely linked to atmospheric oxidative capacity [46]. As the atmospheric environment evolves, the chemical pathways and conversion rates of SO_2_ and NO_2_ may change accordingly. The divergent trends of SOR and NOR highlight that emission reductions alone are not sufficient; the evolving atmospheric chemical environment must also be accounted for to effectively mitigate SIA pollution. Therefore, future control strategies should not only aim to reduce precursor emissions but also consider oxidant levels, humidity, and temperature as important modifiers of secondary aerosol responses. Ignoring these interactions may limit the effectiveness of further emission reductions, especially when traditional precursors are already declining [47].

The SO_4_^2−^/NO_3_^−^ ratio in Guangdong Province exhibited a gradual decline from 1.21 in 2014 to 1.15 in 2024 (Figure 9). This trend indicates a shift in the relative contributions of sulfate and nitrate within SIAs, with nitrate becoming increasingly prominent over time.

### 3.3 Changes in Energy Structure and Its Relationship with Pollutants

**FIGURE 5:**
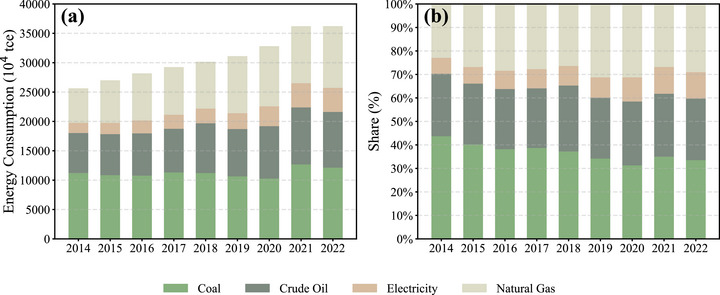
Trends in total energy consumption and relative energy structure in Guangdong Province from 2014 to 2022. (a) Absolute consumption by energy type; (b) proportional share of each energy source. Data represent total primary energy supply. Specifically, electricity refers to the supplied electricity as primary energy and does not include the fuel inputs used for thermal power generation. Coal includes consumption for both power generation and other uses.

The energy structure in Guangdong Province has undergone notable transformations over the past decade. As shown in Figure 5, the total primary energy consumption has exhibited a steadily increasing trend, consistent with the rising GDP of the province [48]. Although total energy consumption continued to increase, the share of coal declined year by year, while the shares of electricity and natural gas gradually rose, indicating a shift in the energy mix toward cleaner sources.

Specifically, the share of coal in primary energy consumption decreased from 43.7% in 2014 to 33.5% in 2022, while the shares of electricity and natural gas rose from 6.8% to 11.3% and from 22.9% to 29%, respectively. This trend is indicative of China's broader shift toward energy electrification [49].

**FIGURE 6:**
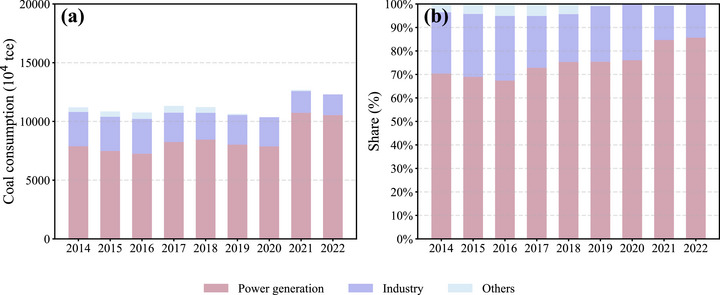
Trends in coal consumption and sectoral distribution in Guangdong Province from 2014 to 2022. (a) Total coal consumption by sector (10^4^ tce); (b) proportional share of coal use in power generation, industry, and other sectors. Data are sourced from the China Energy Statistical Yearbook and represent primary energy supply. Coal use for power generation includes thermal power plants, while industrial coal consumption refers to use in manufacturing processes and heat production.

However, in terms of absolute consumption, coal usage did not decrease but instead showed a slight increase, rising from 1.12 million tons of standard coal in 2014 to 1.21 million tons in 2022. Therefore, the reduction in SO_2_ concentrations cannot be simply attributed to a decrease in total coal consumption. A more detailed analysis reveals that the end use of coal has changed significantly. As illustrated in Figure 6, the proportion of coal used for power generation increased from 70.4% in 2014 to 86.9% in 2022, while coal directly consumed by industry declined from 26.0% to 14.5% during the same period. In other words, although total coal consumption remained relatively constant, a greater share was directed toward electricity generation, and less was directly consumed by industrial sectors. This shift is likely more directly related to SO_2_ reductions than the total volume of coal consumed. Given that coal‐fired power plants are typically subject to stricter flue gas desulfurization (FGD) regulations compared to scattered industrial coal users, this transition likely contributed to a reduction in SO_2_ emissions per unit of coal burned.

To quantify the contribution of sectoral coal consumption to the observed decline in SO_2_ concentrations, the linear attribution model described in Section 2.5 was applied. The model fitting yielded a relative emission factor of 0.0033 for power generation and 0.0144 for industrial coal use, indicating that industrial coal combustion was approximately 4.4 times more polluting than power generation per unit of coal consumed. This is consistent with previous research [50]. Based on the fitted model, we estimate that approximately 75% of the total SO_2_ concentration reduction from 2014 to 2022 can be attributed to reduced industrial coal use, while the remaining 25% is associated with changes in the power sector.

**FIGURE 7:**
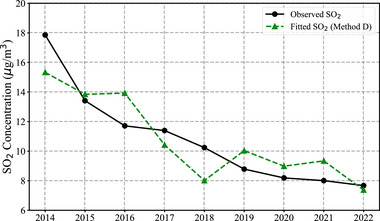
Comparison between observed and fitted SO_2_ concentrations in Guangdong Province from 2014 to 2022.

As shown in Figure 7, the model reproduces the observed SO_2_ concentration trend with reasonable accuracy. While this model does not account for time‐varying emission controls or technology upgrades, it provides a straightforward, data‐driven estimate of sectoral contributions to SO_2_ reductions during the energy transition.

**FIGURE 8:**
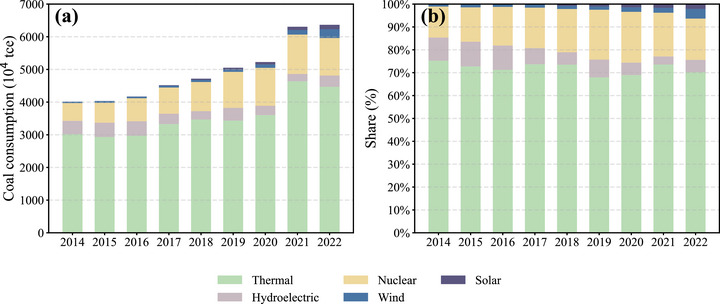
Trends in electricity generation and power structure in Guangdong Province from 2014 to 2022. (a) Annual electricity generation by power source; (b) proportional share of each generation type.

In addition to controlling industrial emissions and improving end‐of‐pipe treatment technologies, optimizing the energy structure through the development of clean energy has become a critical pathway for reducing air pollutant emissions. As shown in Figure 8, between 2014 and 2022, Guangdong Province significantly increased its share of electricity generated from non‐fossil sources (including hydropower, wind, and solar) from 25% to 30%. Among these, solar power exhibited the most rapid growth, expanding from less than 0.2 TWh in 2015 to over 13.4 TWh in 2022. The growth of non‐fossil energy has been driven in part by policies such as the green electricity trading mechanism, which enables large users to purchase certified renewable power.

To further quantify the impact of this transition on ambient SO_2_ concentrations, we conducted a counterfactual analysis using the linear attribution model developed in Section 2.5. Assuming that the non‐fossil electricity in 2022 had instead been generated by coal‐fired power plants, the estimated SO_2_ concentration would have been 2.98 µg/m^3^ higher than observed. Given that the total reduction in SO_2_ concentration from 2014 to 2022 was 10.19 µg/m^3^, the contribution of clean energy development to SO_2_ mitigation is estimated to be approximately 29.3%. These results highlight the significant role that the transformation of the energy system plays in promoting the improvement of regional air quality. These results underscore the importance of clean energy in reducing air pollution. Further expansion of renewables is vital for achieving China's carbon neutrality goals and co‐controlling pollutants such as SO_2_.

Between 2017 and 2023, the number of EVs in Guangdong Province increased from approximately 0.14 million to nearly 3 million, raising the EV share of total vehicles from 0.7% to 9.6%. As shown in Figure S4, this rapid growth reflects the accelerating shift toward low‐emission transportation. During the same period, ambient NO_2_ concentrations dropped from 29.2 to 18.7 µg/m^3^ (Figure 1d). A strong negative correlation was found between EV share and NO_2_ levels (*r* = −0.89), indicating a clear temporal association between transportation electrification and declining NO_2_ concentrations. This pattern is consistent with findings from other urban regions in China, where vehicle electrification has been associated with reduced transportation‐related NO_x_ emission [51].

Nevertheless, NO_2_ originates from multiple sectors, including industry, power generation, and transportation. While the expansion of EVs may have contributed to the decline in transport‐related NO_2_, the overall reduction is the result of multifaceted emission control efforts. However, the present study does not quantify the fraction of additional electricity demand or coal‐fired power generation specifically attributable to EV adoption, so this relationship is discussed here as an association rather than a direct causal attribution. Continued promotion of EVs remains a key strategy for long‐term air quality improvement in China's pathway toward carbon neutrality [52].

These results underscore the importance of an integrated emission control strategy that includes, but is not limited to, vehicle electrification. In particular, increasing the adoption of EVs in both private cars and public transport fleets (e.g., taxis and buses) may deliver co‐benefits for both climate mitigation and public health [53, 54]. Furthermore, ensuring that EVs are supported by an increasingly decarbonized electricity mix will be crucial to maximize their environmental advantage [55, 56].

**FIGURE 9:**
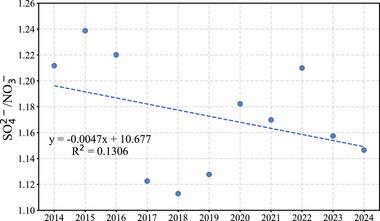
Temporal trend of the sulfate‐to‐nitrate mass ratio (SO_4_^2−^/NO_3_^−^) in Guangdong Province from 2014 to 2024.

To better understand these compositional changes, the evolution of the SO_4_^2^^−^/NO_3_^−^ ratio and aerosol acidity is discussed together below. As shown in Figure 9, the SO_4_^2−^/NO_3_^−^ ratio in Guangdong Province demonstrated an overall decreasing trend from 1.21 in 2014 to 1.15 in 2024, with interannual fluctuations observed throughout the period. This shift in chemical composition indicates an increasing relative contribution of nitrate within SIAs. Meanwhile, concentrations of all three major SIA components (sulfate, nitrate, and ammonium) declined over the same period, reflecting the effects of coordinated emission control policies targeting SO_2_, NO_x_, and NH_3_. Despite these reductions, aerosol pH showed a statistically significant decreasing trend (*p* < 0.05, Figure S5), suggesting that acidification is governed by more complex factors beyond bulk concentrations alone.

Although both sulfate and nitrate are derived from strong acids, their thermodynamic behaviors in the aerosol phase differ considerably. Nitrate is semi‐volatile and contributes to buffering capacity through gas–particle partitioning, whereas sulfate, with limited buffering via bisulfate dissociation, plays a more stable but less adaptive role. Additionally, the ammonium salts that dominate in aerosols have significant interactions with sulfate and nitrate. The thermodynamic properties of ammonium sulfate and ammonium nitrate strongly influence H^+^ activity coefficients (γH^+^), thereby altering the non‐ideality of aerosol acidity. Recent findings by Zheng et al. suggest that nitrate abundance significantly affects γH^+^ and, in turn, modulates the partitioning behavior of ammonium between gas and particle phases. These findings underscore the importance of considering not only the absolute concentrations of acidic and neutralizing species, but also their thermodynamic interactions in determining aerosol acidity. As emission control efforts continue, understanding the coupled behavior of sulfate, nitrate, and ammonium will be essential for predicting future trends in aerosol pH and guiding the development of more effective multipollutant control strategies.

## 4 Conclusions

This study provides a comprehensive assessment of the temporal evolution of PM_2.5_ and its chemical composition in Guangdong Province from 2014 to 2024. We found that SIAs, including sulfate, nitrate, and ammonium, were the main contributors to the decline in PM_2.5_ mass. In particular, sulfate responded more directly to reductions in SO_2_ emissions, whereas nitrate showed a relatively slower response to decreases in NO_2_ emissions. The increasing prominence of nitrate and the gradual decline in the SO_4_^2−^/NO_3_^−^ ratio reflect not only changes in precursor emissions but also evolving atmospheric chemical regimes. Furthermore, although all major acidic and neutralizing components decreased, aerosol acidity continued to rise, suggesting that non‐ideal thermodynamic interactions, particularly involving ammonium and nitrate, played an increasingly important role. The transition in the energy‐use structure, particularly the shift from industrial coal consumption to cleaner electricity, was an important driver of the reduction in atmospheric SO_2_ concentrations, while the decline in NO_2_ was temporally associated with transportation electrification and broader emission control efforts.

These findings suggest that to sustain future improvements in air quality, management strategies should go beyond emission controls alone and adopt an integrated approach that combines end‐of‐pipe measures with systematic structural transitions, such as accelerating the deployment of clean energy, promoting transport electrification, and gradually phasing out high‐emitting industrial processes. Moreover, air pollution control policies should incorporate evolving atmospheric chemistry, including oxidation capacity and thermodynamic interactions, to better anticipate the response of secondary aerosols. As air pollutant and carbon emissions are increasingly intertwined, co‐optimizing pollution and climate strategies will be critical for achieving both clean air and carbon neutrality targets.

## Author Contributions

**Haohao Wang**: conceptualization, data curation, methodology, writing – original draft, supervision, project administration, funding acquisition. **Qing Chen**: conceptualization, visualization, software, writing – review and editing. **Kangan Shu**: visualization, investigation, methodology. **Jinqing Luo**: writing – review and editing, investigation. **Liu Yang**: data curation, writing – review and editing.

## Conflicts of Interest

The authors declare no conflict of interest.

## Supporting information




**Supporting File**: gch270108‐sup‐0001‐SuppMat.docx.

## Data Availability

The data that support the findings of this study are openly available in Zenodo at https://doi.org/10.5281/zenodo.19382405, reference number https://doi.org/10.5281/zenodo.19382405.
