# Supplementary material for: Decadal Evolution of Atmospheric Sulfate and Nitrate and Its Potential Link to Energy Restructuring: A Case Study in Guangdong Province, China
Source: Glob Chall. 2026 Apr 25;10(4):e70108. doi: 10.1002/gch2.70108 (PMC13109783; doi:10.1002/gch2.70108)
Supplement: Supplementary file 1 — Supporting File: gch270108‐sup‐0001‐SuppMat.docx. [file GCH2-10-e70108-s001.docx]

Decadal evolution of atmospheric sulfate and nitrate and its potential link to energy restructuring: A case study in Guangdong Province, China

**Supplementary Information**

**Contains 7 pages, 8 figures**

1.It is noteble that significant spatial heterogeneity was observed in pollutant decline rates across cities in Guangdong Province. As illustrated in Figure S2, the province was divided into the Pearl River Delta (PRD) region and non-PRD areas (including eastern, western, and northern Guangdong), based on administrative boundaries and development level. In general, cities within the PRD region exhibited faster reductions in key pollutants such as PM_2.5_ and SO_2_ compared to cities in non-PRD areas (Figure S3). On average, the PM_2.5_ the relative reduction rate in PRD cities was 44.5% from 2014 to 2024, compared to 38.42% in non - PRD cities. Similarly, SO_2_ relative reduction rate in PRD cities was 62.58%, significantly higher than the 56.35% in non-PRD areas. NO_2_ also showed a slightly faster decline in PRD cities, with a relative reduction rate of 30.46%, compared to non-PRD cities (27.41%), although the difference was smaller compared to particulate pollutants.

Among individual cities, Zhuhai, Guangzhou, and Foshan achieved the largest PM_2.5_ reductions within the PRD, exceeding 18% over the study period, while cities such as Zhaoqing and Huizhou exhibited relatively slower improvements. In non-PRD regions, the declines were more modest. Cities like Meizhou and Shaoguan achieved moderate reductions (~17–18%), whereas others such as Zhanjiang and Shanwei saw much smaller improvements, with PM_2.5_ reductions of less than 5%.

Regarding ozone (O_3_), although both PRD and non-PRD cities exhibited decreasing trends on average (the relative reduction rate being 39.7% in PRD and 33.40% in non-PRD), substantial inter-city variability was observed. Notably, Dongguan and Guangzhou achieved considerable O_3_ reductions (>40%), while some cities such as Zhanjiang and Yangjiang showed only marginal decreases or even slight stagnation. These findings are consistent with previous studies emphasizing the challenge of controlling ozone pollution in VOC-rich and highly urbanized environments^[1–4]^.

Meteorological conditions, including temperature, relative humidity, precipitation, and wind speed, were also analyzed over the study period to assess potential background variability (Figure S4). The results showed that the annual average temperature exhibited a slight increasing trend from 2014 to 2024. In contrast, relative humidity and precipitation levels remained relatively stable, with no clear upward or downward trends. Wind speed showed minor fluctuations year-to-year but did not present a significant long-term trend either.


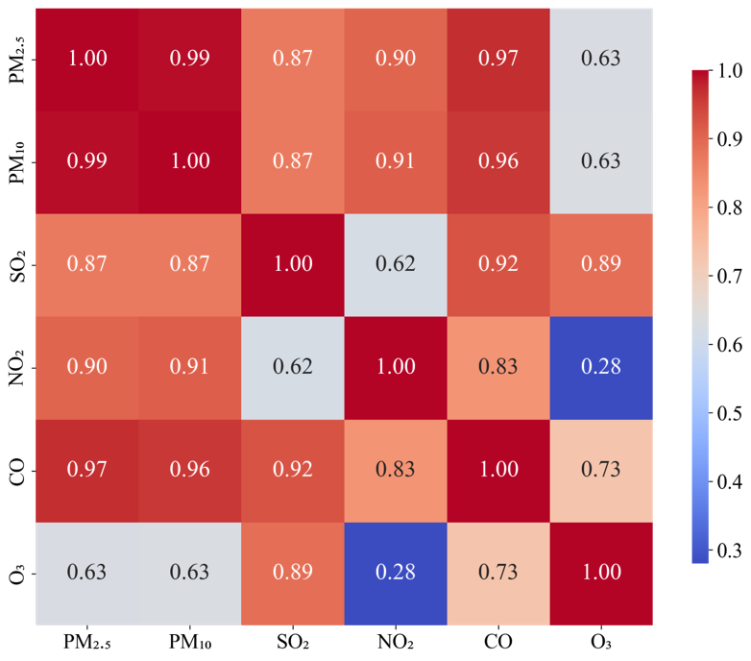


**Figure S1** Correlation matrix of annual average concentrations of major air pollutants from 2014 to 2024.


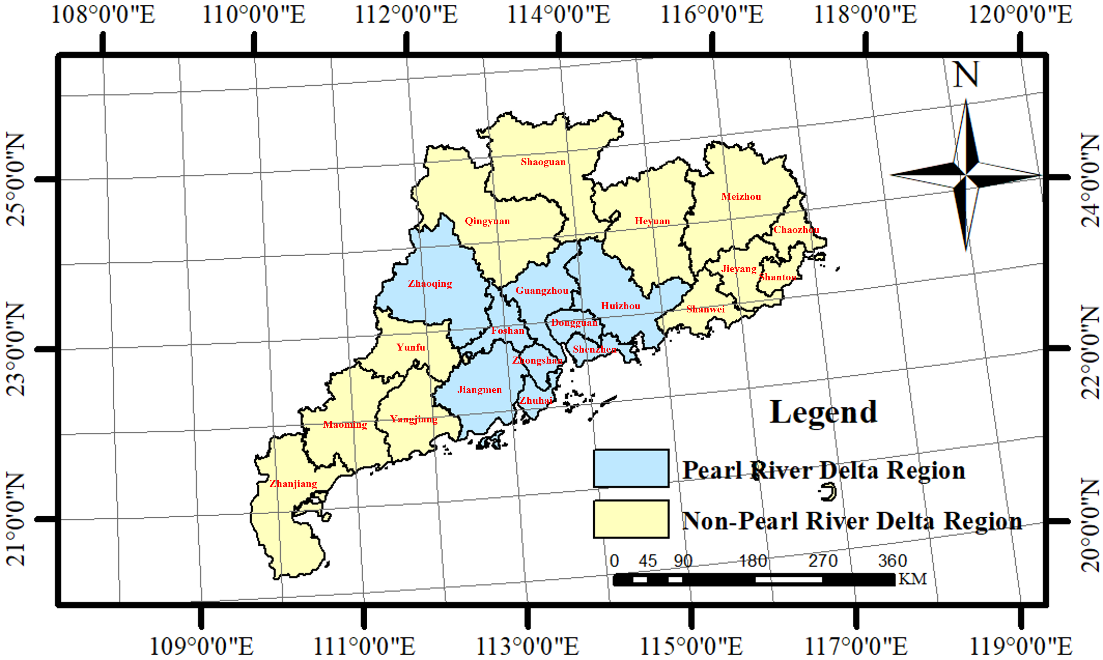


**Figure S2** Geographical division of the Pearl River Delta and non-Pearl River Delta regions in Guangdong Province.


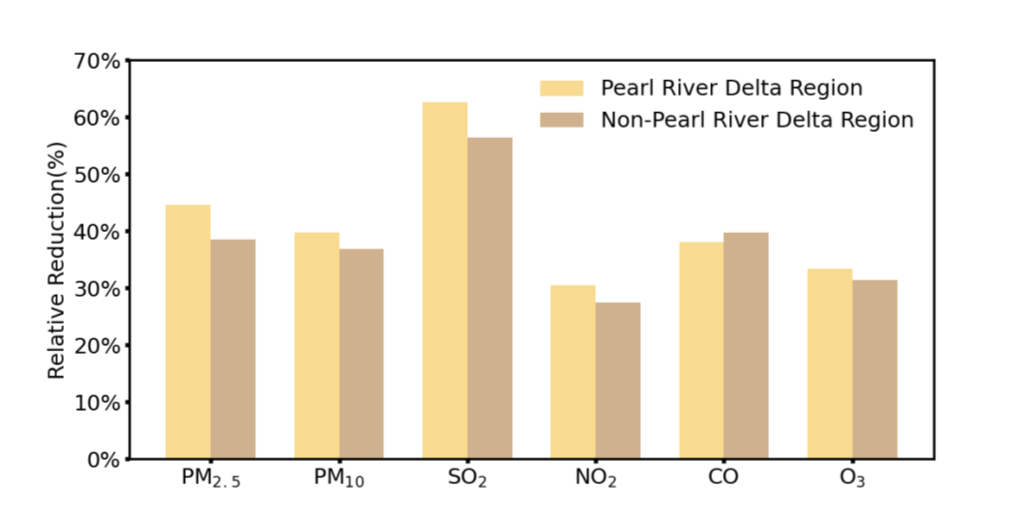


**Figure S3** Comparison of relative reduction of major air pollutants from 2014 to 2024 in the Pearl River Delta and non-Pearl River Delta regions.


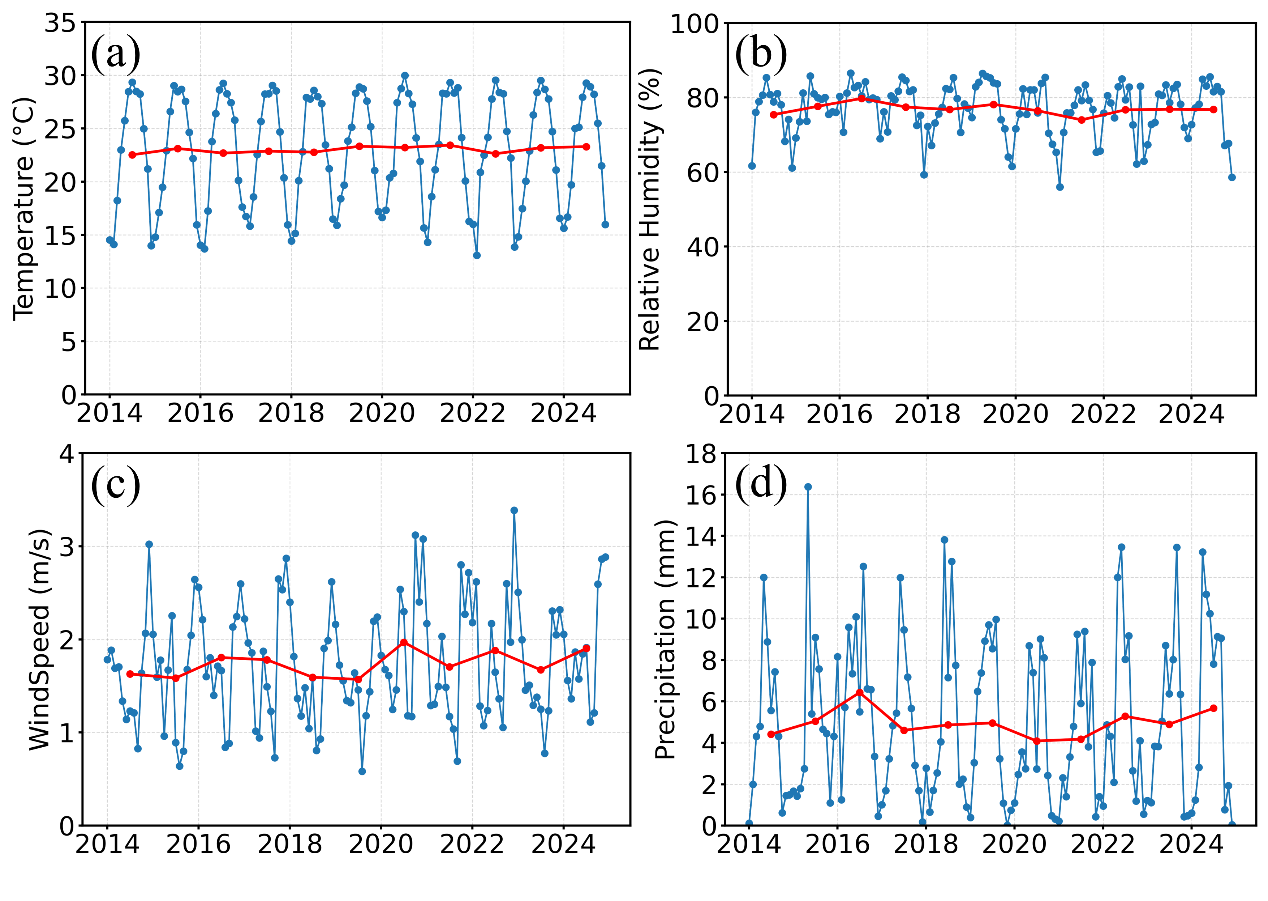


**Figure S4** Temporal variation of meteorological parameters from 2014 to 2024 in Guangdong Province. (a) Temperature; (b) relative humidity; (c) wind speed at 10 m; (d) precipitation. Data were obtained from the ERA5 reanalysis dataset provided by the European Centre for Medium-Range Weather Forecasts (ECMWF).


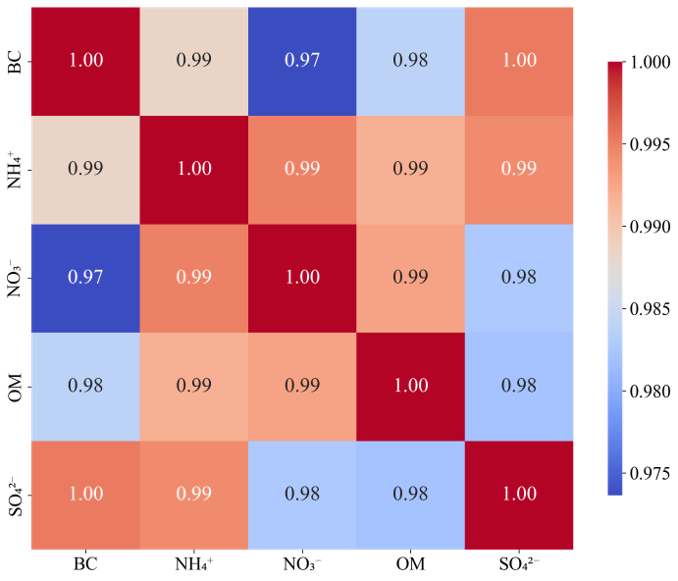


**Figure S5** Correlation matrix of major PM_2.5_ chemical components in Guangdong Province from 2014 to 2024. The matrix shows Pearson correlation coefficients among five major components: black carbon (BC), ammonium (NH_4_^+^), nitrate (NO_3_^-^), organic matter (OM), and sulfate (SO_4_^2-^), based on monthly mean concentrations.


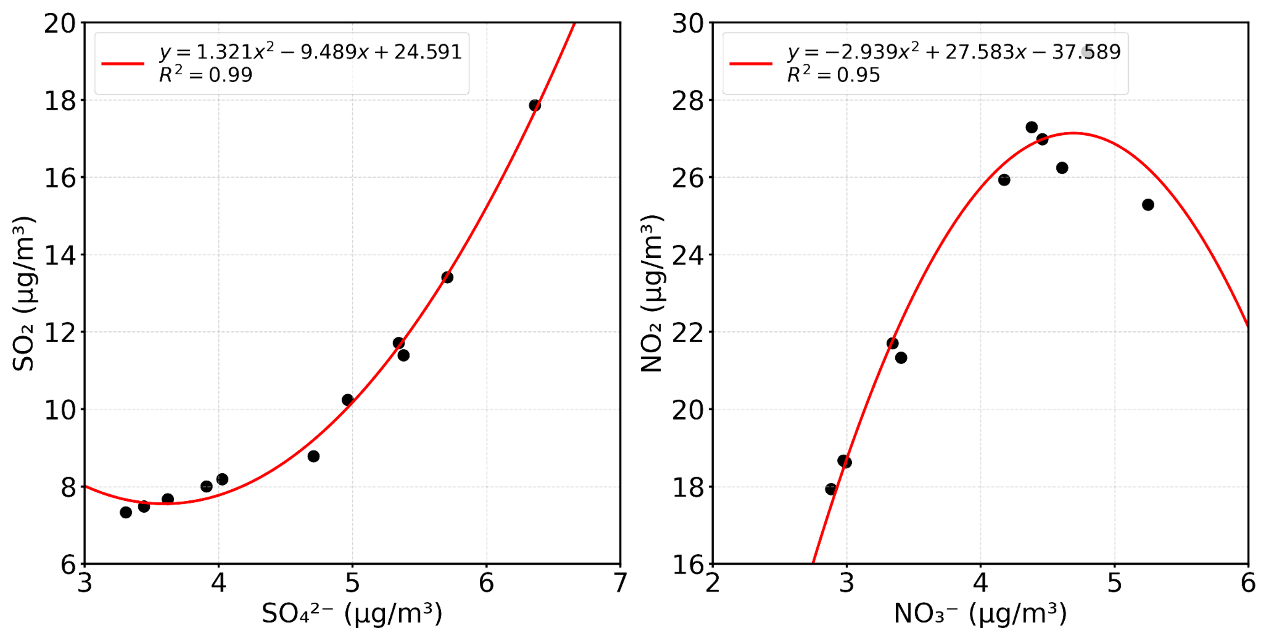


**Figure S6** Nonlinear relationships between secondary inorganic aerosols and their precursors in Guangdong Province.


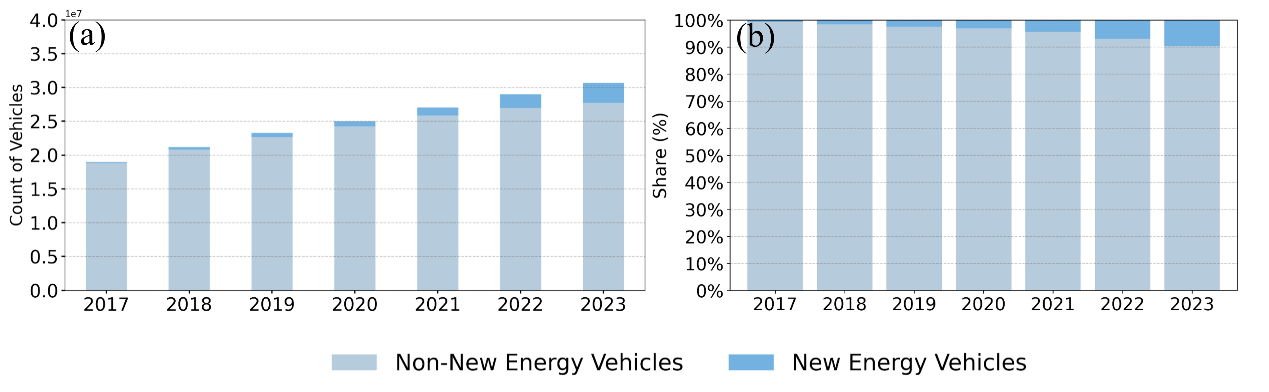


**Figure S7** Trends in vehicle stock and the share of new energy vehicles (NEVs) in Guangdong Province from 2017 to 2023. (a) Total number of vehicles by category; (b) proportion of new energy vehicles.


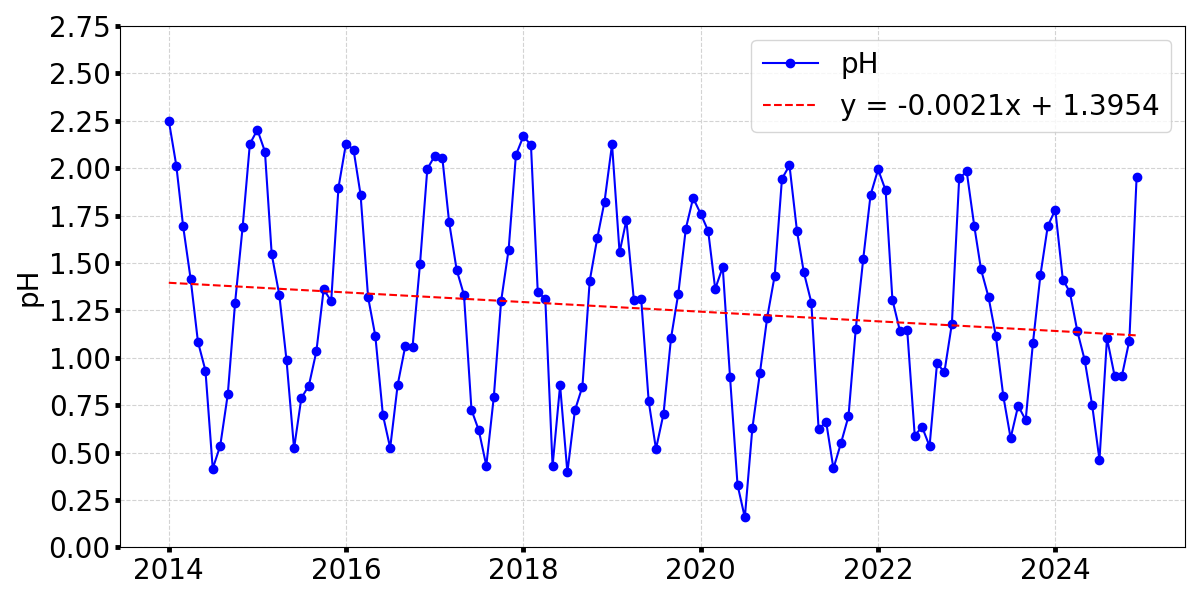


**Figure S8** Monthly mean aerosol acidity (pH) in Guangdong Province from 2014 to 2024.The blue line indicates monthly mean pH values, while the red dashed line shows the linear trend fitted to the entire period. A decreasing trend suggests a gradual acidification of aerosol particles over the past decade.

**References**

[1] Liu C, Lu B, Wang Q, *et al.* (2024) High-level HONO exacerbates double high pollution of O_3_ and PM_2.5_ in China. *Science of The Total Environment* **945**, 174066.

[2] Zhao M, Zhang Y, Pei C, *et al.* (2022) Worsening ozone air pollution with reduced NO and VOCs in the Pearl River Delta region in autumn 2019: Implications for national control policy in China. *Journal of Environmental Management* **324**, 116327.

[3] Zhang X, Stocker J, Johnson K, *et al.* (2022) Implications of Mitigating Ozone and Fine Particulate Matter Pollution in the Guangdong‐Hong Kong‐Macau Greater Bay Area of China Using a Regional‐To‐Local Coupling Model. *GeoHealth* **6**, e2021GH000506.

[4] Wang T, Xue L, Brimblecombe P, *et al.* (2017) Ozone pollution in China: A review of concentrations, meteorological influences, chemical precursors, and effects. *Science of The Total Environment* **575**, 1582–1596.
